# Production of high titer of citric acid from inulin

**DOI:** 10.1186/s12896-019-0503-0

**Published:** 2019-02-11

**Authors:** Magdalena Rakicka, Jakub Wolniak, Zbigniew Lazar, Waldemar Rymowicz

**Affiliations:** Department of Biotechnology and Food Microbiology, Wroclaw University of Environmental and Life Sciences, 37 Chełmońskiego St, 51-630 Wroclaw, Poland

**Keywords:** Inulin, Citric acid, Repeated-batch culture, *Yarrowia lipolytica*

## Abstract

**Background:**

Citric acid is considered as the most economically feasible product of microbiological production, therefore studies on cheap and renewable raw materials for its production are highly desirable. In this study citric acid was synthesized by genetically engineered strains of *Yarrowia lipolytica* from widely available, renewable polysaccharide – inulin. Hydrolysis of inulin by the *Y. lipolytica* strains was established by expressing the inulinase gene (*INU1* gene; GenBank: X57202.1) with its native secretion signal sequence was amplified from genomic DNA from *Kluyveromyces marxianus* CBS6432. To ensure the maximum citric acid titer, the optimal cultivation strategy–repeated-batch culture was applied.

**Results:**

The strain *Y. lipolytica* AWG7 INU 8 secreted more than 200 g dm^− 3^ of citric acid during repeated-batch culture on inulin, with a productivity of 0.51 g dm^− 3^ h^− 1^ and a yield of 0.85 g g^− 1^.

**Conclusions:**

The citric acid titer obtained in the proposed process is the highest value reported in the literature for *Yarrowia* yeast. The obtained results suggest that citric acid production from inulin by engineered *Y. lipolytica* may be a very promising technology for industrial citric acid production.

## Background

Production of citric acid (CA), the intermediate of Krebs cycle, is one of the oldest technology of organic acid production applied at industrial scale [1]. Initially CA was extracted from Italian lemons which what was later replaced by its biosynthesis using *Aspergillus niger* [2]. Afterwards, the discovery of CA secretion by the yeast *Yarrowia lipolytica* caused a rapid progress in research on that process [3]. The global CA production in 2015 reached 2 Mln tones and 3.7% of annual growth is expected till 2020 (https://ihsmarkit.com/products/citric-acid-chemical-economics-handbook.html). Therefore, CA is the most abundantly produced chemical on an industrial scale and the most widely used organic acid, it has also “generally regarded as safe” (GRAS) status [4]. The main function of this acid is its application as food acidulant. It is also used for prevention of oxidative deterioration in flavor or color [5]. CA and its derivatives are also ingredients in detergents, personal care products as well as they are widely used in pharmaceutical and biomedical industry. Due to many applications of this valuable compound, there is a great demand on developing very efficient technology for its production. Nowadays, from an industrial point of view, the most important challenges when developing novel technologies are to design the production process with high titer, productivity and yield, simultaneously fulfilling the main principles of cleaner production, energy saving and sustainable development [6].

One of the criteria when developing a new biotechnology process is the cultivation system. The most common systems used industrially are batch or fed-batch cultures. To achieve better results, the repeated-batch culture (RBC) could be used. This cultivation system allows for better dynamics and higher efficiency of the biosynthesis process by extending the effective production phase in comparison to traditional batch culture [7]. The RBC was already successfully used for lipid, ethanol and erythritol production [7–9].

Other important criterion of efficient technology using microorganisms is the substrate applied in the process. The raw materials used for biotechnological processes must be cheap, renewable and not competing with food production. Ideally, such a feedstock should be waste or by-products from other industries.

The nonconventional *Y. lipolytica* yeast is rapidly emerging as a valuable host for the production of a variety of lipids, organic acids, polyols and other metabolites [10]. Nowadays, both, lab-scale as well as industrial production of chemicals using *Y. lipolytica*, are being improved through genetic engineering. Recently, Sabra et al. [11] focused on transcriptome and fluxome characterization of citrate producing strain ACA DC 50109 and enhanced citrate secretion in a glucose based medium to 55 g dm^− 3^. Furthermore, improvement of succinic acid production (110.7 g dm^− 3^) was achieved through deletion of succinate dehydrogenase and CoA-transferase genes followed by overexpression of TCA cycle genes [12]. More recent work characterized biological pathway of erythritol biosynthesis and improved its production through overexpression of transketolase or erythrose reductase genes in *Y. lipolytica* [13, 14]. Furthermore, β-carotene production in *Y. lipolytica* was also investigated [15]. Overexpression of the optimum promoter-gene pairs for each transcriptional unit in lipid overproducing strain followed by optimization of cultivation method significantly improved β-carotene production in comparison to strain without lipid overproduction.

In the processes of CA production using *Y. lipolytica* many research groups focused on glycerol, by-product from biodiesel or soap production [16–18]. However, some other waste substrates were also applied: olive mill waste-water [19], molasses [20] or pretreated cellulose [21]. The plant biomass constitutes very promising raw material due to high sugar content and its availability. Besides cellulose, some plants, such as chicory, Jerusalem artichoke or dahlia, are known to store energy in the form on inulin (IN), a fructose polymer, which is accumulated in large quantity in roots and rhizoids [22]. Inulin is classified as dietary fibre and promotes the growth of intestinal bacteria [23]. IN has significant number of pharmaceutical and food applications. It is frequently used as sugar or fat substitute in different types of food and as an excipient and stabilizer in many pharmaceuticals [24, 25]. Furthermore, IN was found to have also anti-cancer [26] and immuno-modulatory properties [27]. The global IN market was valued at USD 1674.3 Mln in 2017, and is expected to reach USD 5099.2 Mln by 2025. Interestingly, Europe is the largest IN market which accounts for approximately 42% of the global IN market (http://www.acutemarketreports.com/report/artichoke-inulin-market-professional-survey-report). Instantly growing IN market results in increasing amounts of inulin-rich wastes after its production that must be managed. One of the possibilities to utilize these raw materials is microbiological production of bioethanol, single-cell protein, lipids, CA, butanediol or lactic acid [28–32].

Due to the always high demand for CA and increasing market of IN, the main goal of this study was to improve biosynthesis of CA by engineered *Y. lipolytica* strains, using IN as carbon source. To enhance the CA titer, yield and productivity from IN, repeated-batch culture was applied.

## Results

### Overexpression of *INU1* gene in *Y. lipolytica* strains

**Fig. 1:**
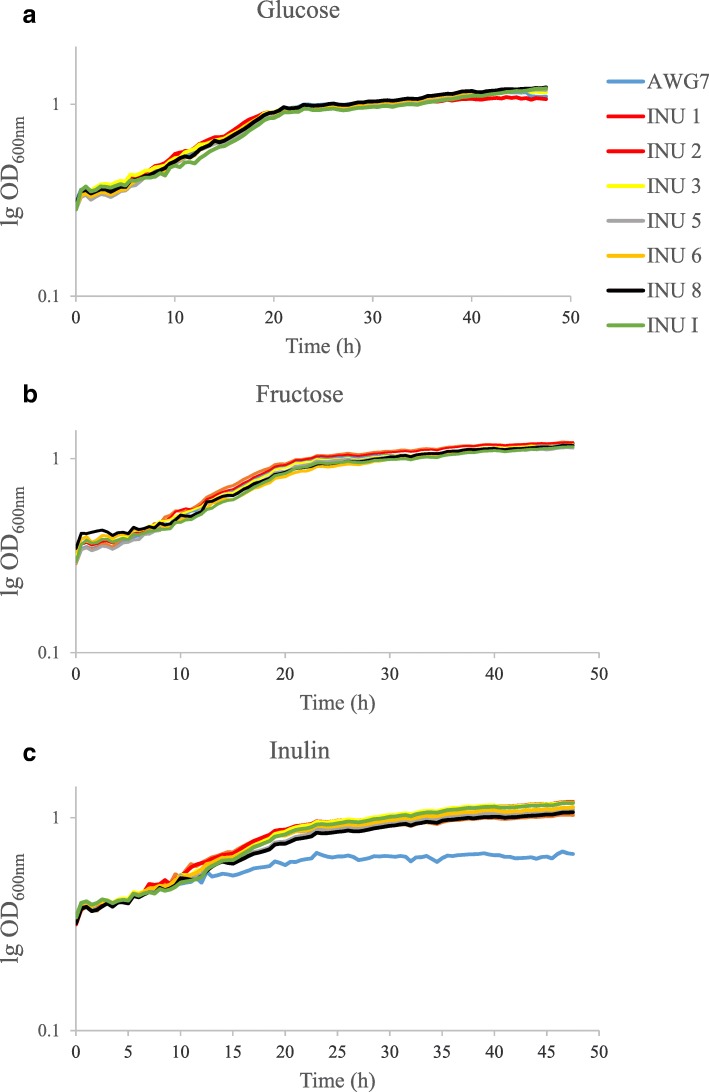
Growth curves of various *Y. lipolytica* strains growing on glycose (**a**), fructose (**b**) and inulin (**c**): AWG7 (blue line), AWG7 INU 1 (dark blue line), AWG7 INU 2 (red line), AWG7 INU 3 (yellow line), AWG7 INU 5 (grey line), AWG7 INU 6 (orange line), AWG7 INU 8 (black line), AWG7 INU I (green line). The strains were grown on an YNB/glucose, fructose or inulin medium. Quintuple experiments were performed at 28 °C under constant agitation using Bioscreen C (Oy Growth Curves Ab Ltd.)

It is known phenomenon, that CA secretion by *Y. lipolytica* occurs when nitrogen and to some level phosphorous are deficient in the medium. The abilities to secrete large amount of this acid are also strain dependent [33–35]. In the current study, new inulinase expressing derivatives of *Y. lipolytica* AWG7, able to grow on IN, were investigated for CA production from this raw material. The parental AWG7 strain was selected as efficient CA producer, with high purity of CA over ICA. During continuous culture with glycerol as carbon source, it secreted over 97 g dm^− 3^ of CA from 150 g dm^− 3^ of glycerol [36]. In the current study, growth of the wild strain and its inulinase positive derivatives was analyzed in YNB medium supplemented with fructose or IN (Fig. 1). Furthermore, control experiment with glucose was also performed to analyze if the random *INU1* gene integration into *Y. lipolytica* genome, did not affect growth of the resulted transformants (Fig. 1). No difference was observed for all analyzed strains in both, YNB medium with glucose and fructose (Fig. 1a, b). Although the integration cassette for *INU1* gene expression was inserted into the genome using retrotransposable zeta sequences [37], what causes randome integration and may result in different inulinase expression, no differences was observed among the analyzed transformants growing in medium with IN (Fig. 1c). Slow and not very significant growth of the wild strain AWG7 may be the result of trace amounts of free fructose present in the medium (Fig. 1c). The good growth of all analyzed transformants in medium with IN is an indirect proof for efficient inulinase expression and hydrolysis of IN. Similar growth pattern for all strains was the basis for their further analysis for CA biosynthesis.

### Selection of the best CA producer using batch cultures

In the next step of the study, all the engineered strains were analyzed for their abilities to secrete CA while growing in medium with IN. In control experiment, the AWG7 strain grew in synthetic medium with fructose and glucose. The obtained results are presented in Table 1. Inulin concentration of 100 g dm^− 3^ was used at the beginning of the process. The culture was carried out until the available substrate (fructose released from inulin) was exhausted from the medium. Fructose concentration was monitored by HPLC method. Six out of seven analyzed transformants were able to secrete more than 60 g dm^− 3^ of CA from 100 g dm^− 3^ of IN. The best CA producing strain - AWG7 INU8 - secreted 75.5 g dm^− 3^. It is very important to notice, that parental AWG7 strain secreted only 48.7 g dm^− 3^ of CA in fructose based medium, which was significantly lower not only compared to glucose based medium but also to all its derivatives growing in medium with IN (fructose polymer). What is also important to mention is the fact that the concentration of the undesirable ICA remained for all transformants at very low level (less than 2 g dm^− 3^). The processes using different inulinase expressing strains differed significantly in terms of CA productivity and yield. While the best CA producer secreted this acid with the productivity of 0.8 g dm^− 3^ h^− 1^, the highest value of this parameter was reached by AWG7 INU5 strain (0.93 g dm^− 3^ h^− 1^). Additionally, although the CA yield was very different depending on the strain, all the obtained values were satisfactory, reaching 0.76 g g^− 1^ for AGW7 INU 8 strain. This value was 35% higher than the CA yield obtained by the parental AWG7 strain on fructose. In the process with IN as carbon source some of the analyzed strains produced also small amounts of mannitol into the culture broth, however, concentration of this by product remained at low level and did not exceed 7 g dm^− 3^.

**Table 1:** Parameters of citric acid synthesis by various *Y. lipolytica* strains in batch culture

Strain		*Yarrowia lipolytica*					
	Substrat	Biomass	Citric acid	Isocitric acid	Mannitol	Q_CA_	Y_CA_
		g dm^− 3^				g dm^− 3^ h^− 1^	g g^− 1^
AWG7 Control 1	Fructose	12.0 ± 0.8	48.7 ± 2.0	2.0 ± 0.4	1.1 ± 0.2	0.42 ± 0.7	0.49 ± 0.01
AWG7 Control 2	Glucose	13.8 ± 1.0	72.9 ± 0.8	2.5 ± 0.9	0.0	0.83 ± 0.5	0.73 ± 0.01
AWG7 INU 1	Inulin	9.0 ± 2.0	64.6 ± 1.6	1.4 ± 0.2	6.6 ± 1.1	0.72 ± 0.9	0.64 ± 0.02
AWG7 INU 2	Inulin	10.5 ± 0.8	71.6 ± 0.9	1.4 ± 0.5	5.3 ± 1.0	0.79 ± 0.4	0.72 ± 0.02
AWGZ INU 3	Inulin	12.0 ± 0.6	69.2 ± 1.4	1.4 ± 0.7	4.1 ± 0.4	0.72 ± 0.5	0.70 ± 0.01
AWG7 INU I	Inulin	11.0 ± 1.1	58.0 ± 4.1	1.4 ± 0.5	0.0	0.50 ± 0.7	0.60 ± 0.02
AWG7 INU 5	Inulin	13.0 ± 0.9	65.6 ± 1.8	1.4 ± 0.5	3.7 ± 0.4	0.93 ± 0.3	0.66 ± 0.01
AWG7 INU 6	Inulin	16.5 ± 1.2	64.6 ± 2.8	1.4 ± 0.4	6.1 ± 1.3	0.88 ± 0.5	0.65 ± 0.02
AWG 7 INU 8	Inulin	15.8 ± 0.9	75.5 ± 1.5	1.5 ± 0.4	0.0	0.80 ± 0.7	0.76 ± 0.02

*Q*_*CA*_ productivity of citric acid, *Y*_*CA*_ yield of citric acid

Significant differences were also observed among the analyzed strains in terms of biomass production. The concentration of cells in the culture ranged from 9.0 g dm^− 3^ to 16.5 g dm^− 3^. The parental AWG7 strain grew only slightly better in medium with glucose compared to fructose based medium.

### RBC as a toll of process intensification

**Fig. 2:**
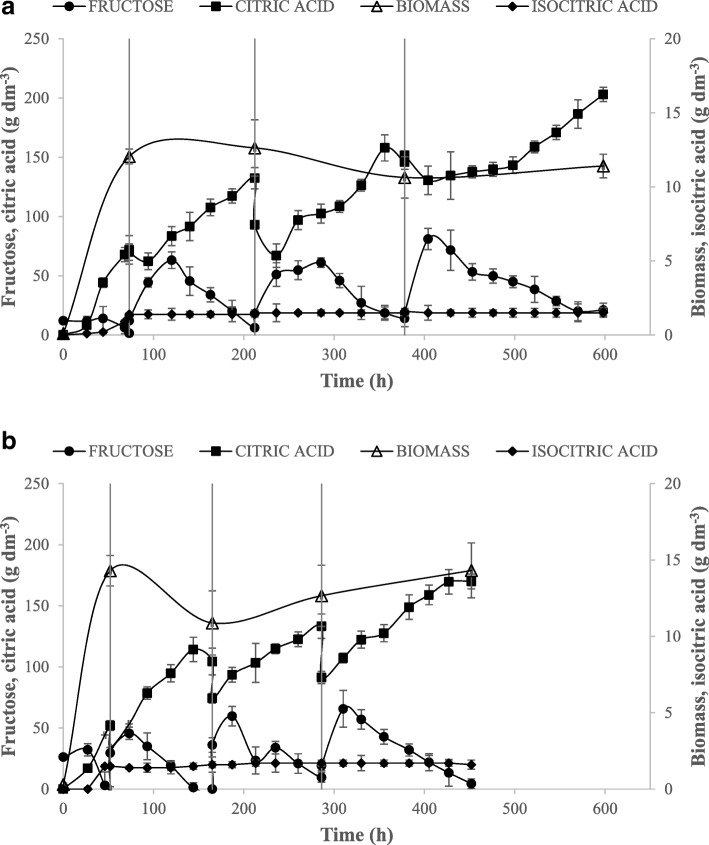
Time courses of biomass production (Δ), citric acid (■) and isociric (♦) acids titer and fructose consumption (●) during repeated batch culture of AWG7 INU 8 (**a**) and AWG7 INU 5 (**b**) strains of *Y. lipolytica*. 40% of medium was replacement for four times. Each variant of RBC was repeated two times

**Fig. 3:**
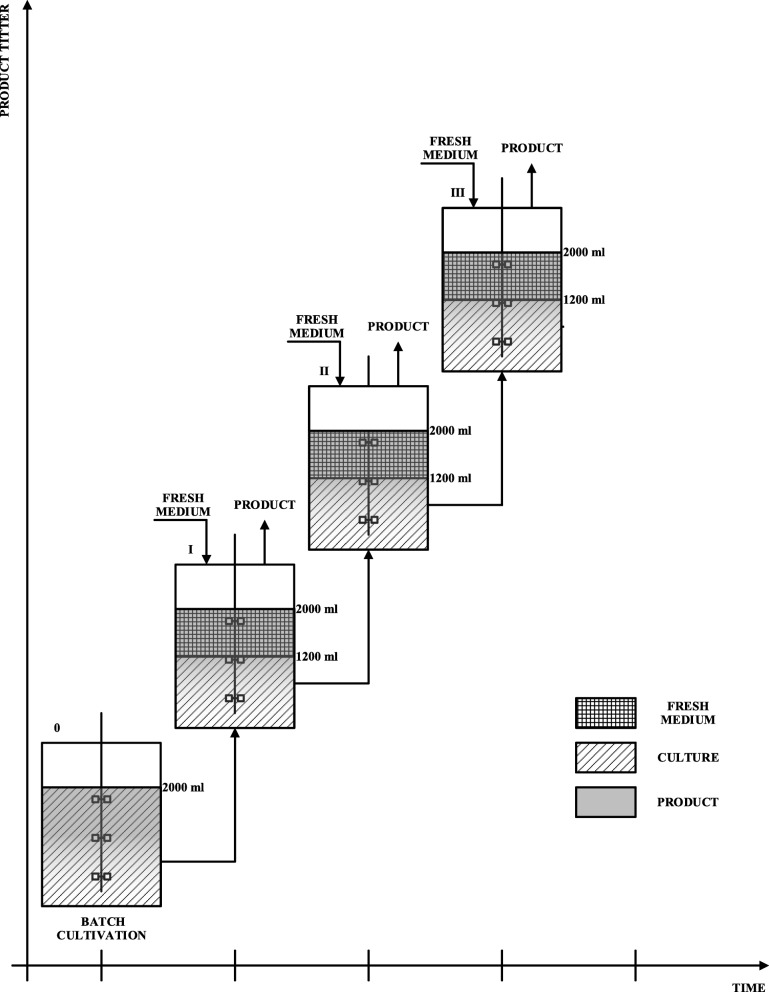
The scheme of increasing product titer during the repeated-batch culture used in this study

Two best CA producers identified in the previous stage of the study was chosen for further analysis. The AWG7 INU8 transformant was used due to its highest CA yield and AWG7 INU5 strain was chosen due to its highest CA productivity. Both strains were cultured using RBC system to increase the titer, yield and productivity obtained during traditional batch cultures. The kinetics of the RBC processes (changes of biomass, CA and fructose over time) for both transformants was presented on Fig. 2. There was no technical difficulties observed during the quintuple RBC experiment as well as no microbiological contamination occurred. CA biosynthesis using this system is stable and feasible new process. The whole process remained stable during 450 to 600 h. Medium exchange in the bioreactor (40% of working volume each time) was carried out four times (Figs. 2 and 3). The rate of medium exchange was chosen based on the previous work on erythritol production in RBC system [6].

The RBC processes for both strains differed slightly in terms of biomass biosynthesis (Fig. 1). Higher concentration of cells was observed for AWG7 INU5 strain (Fig. 1b), however, the biomass concentration dropped after the first medium exchange for both strains (Fig. 1). IN hydrolysis was occurred rapidly in both processes which can be seen as high increase of fructose concentration in the medium. Higher fructose utilization rate was reached by AWG7 INU5 strain (Fig. 1b) which resulted in 150 h shorter process compared to AWG7 INU8 transformant (Fig. 1a). In contrary, AWG7 INU8 strain secreted 30 g dm^− 3^ more of CA into the medium (203 g dm^− 3^) compared to AWG7 INU5 strain (170 g dm^− 3^). Furthermore, after two medium exchange, the concentration of CA was increasing for both strains.

The overall CA yield of the RBC process was higher for AWG7 INU 8 strain and reached 0.51 g g^− 1^ (Table 2). Albeit, the overall CA yield did not obtain very high values, the maximum partial yield reached 0.85 g g^− 1^ for AWG7 INU 8 strain in 2nd exchange of the medium (Table 2). The overall CA productivity was almost the same for both strains and reached 0.3 g dm^− 3^ h^− 1^ (Table 2). The highest CA productivity (0.51 g dm^− 3^ h^− 1^) during the RBC process was noted for AWG7 INU8 strain during the 2nd medium exchange (Table 2). The concentration of the undesired ICA during the whole RBC process for both transformants did not exceed 2 g dm^− 3^ (Table 2).

**Table 2:** Citric acid productivity and yield from inulin for *Y. lipolytica* strains expressing inulinase gene in repeated batch culture

Strain	Q_ov_	Q_H_	Y_ov_	Y_H_
	(g dm^− 3^ h^− 1^)		(g g^− 1^)	
AWG7 INU8	0.34 ± 0.02	0.51 ± 0.04	0.51 ± 0.02	0.85 ± 0.03
AWG7 INU5	0.37 ± 0.08	0.49 ± 0.07	0.42 ± 0.01	0.61 ± 0.01

*Q*_*ov*_ overall productivity of citric acid production, *Y*_*ov*_ overall yield of citric acid production during RBC, *Q*_*H*_ the highest productivity noticed during the process, *Y*_*H*_ the highest yield noticed during the process

### Activity of inulinase displayed on the yeast cells

**Fig. 4:**
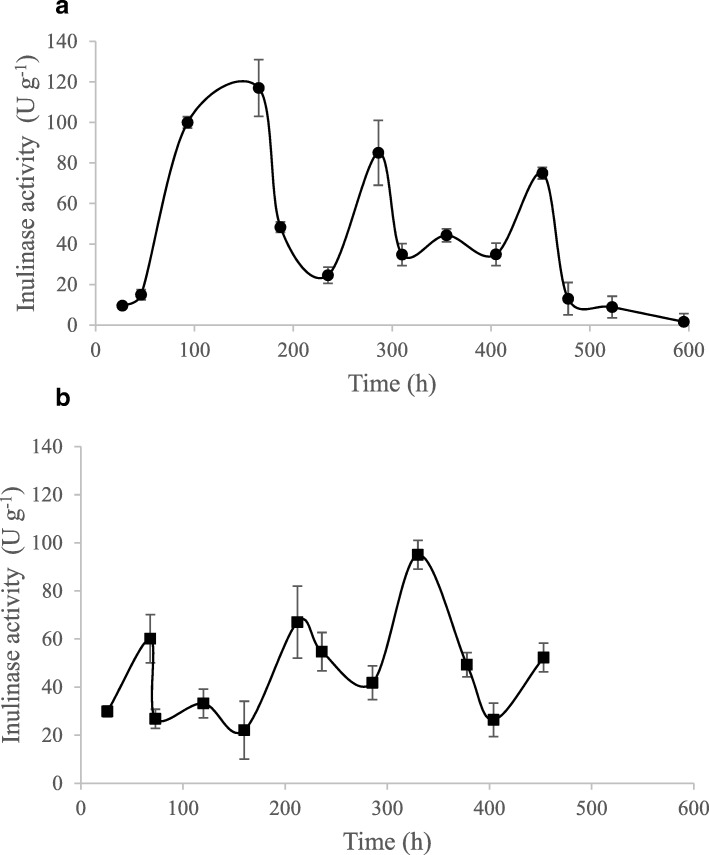
Time course of inulinase production by *Y. lipolytica* AWG7 INU 8 (**a**) and AWG7 INU5 (**b**) transformants during repated-batch culture on inulin

During RBC on IN, the activity of inulinase was measured. Both strains showed the activity of expressed enzyme (Fig. 4). The initial activity of inulinase was increased until the first replacement of the medium was done for both strains (Fig. 4) and then decreased when concentration of released fructose was decreased. The highest inulinase activity was noted for AWG7 INU 8 strain which reached 120 U g^− 1^ of cell dry mass (Fig. 4a). For AWG7 INU5 strain maximum activity of inulinase was reached after 320 h of the culture (Fig. 4b).

## Discussion

The CA is mostly produced organic acid using from molasses and fungi *A. niger.* Although the process is well known and used for decades, it shows some disadvantages which must be eliminated – it is multi-stages and dependent on the purity of the substrate. Therefore, the whole process generates some environmentally undesired wastes [17]. As shown previously, CA biosynthesis using *Y. lipolytica* may become an efficient alternative method if its production [36, 38]. The process with yeast is more favorable due to the higher resistance of the cells to high substrate concentration and their high resistance to metal ions. Furthermore, yeast are easy to culture and to establish stable continuous processes of desired metabolites production. Additional advantage of using yeast, especially *Y. lipolytica*, is the availability of genetic tools to engineer their metabolism and boost the production processes [39].

The CA production by *Y. lipolytica* were based mainly on glucose and crude glycerol [33, 36, 40, 41]. However, the global increase in IN and prebiotic food manufacturing caused growing interest in utilization of IN for biotechnological processes which may help to reduce the amount of IN-rich wastes generated during the production process [30, 31, 42]. Application of IN for the processes with *Y. lipolytica* in concert with the use of RBC system could help to develop more efficient CA production technology. The use of the RBC system for CA biosynthesis is a very desirable solution. It offers application of high concentrated media without inhibition of cells growth due to the high concentration of biomass in the bioreactor. Furthermore, the secreted acid is partially removed from the tank which eliminated its toxicity to the cells.

In the literature, many examples of CA production by *Y. lipolytica* using different cultivation systems can be found. Summary of the available results is presented in Table 3. The highest CA titer was obtained in the presented work by *Y. lipolytica* AWG7 INU 8 transformant during RBC process with inulin. The CA titer obtained by AWG7 INU 5 strain is also higher to these obtained for other strains growing on sunflower oil using batch cultures or on glycerol using fed-batch systems [16, 43, 44]. Furthermore, studies with the parental AWG7 strain using RBC system with glycerol [44] also did not reach the concentration of CA obtained by its inulinase positive transformants studied in the current work (Table 3). Nevertheless, the parental strain used in the previous work presented higher productivity during the RBC process [44]. In the study conducted by Liu et al. [42], genetic modified strain of *Y. lipolytica* growing on inulin in batch culture, produced CA almost with the same yield (0.84 g g^− 1^) as the strain AWG7 INU8 used in this study in RBC system.

**Table 3:** Citric acid production by various strains of *Yarrowia lipolytica*

Strain	System	Substrate	Citric acid (g dm^−3^)	Q (g dm^− 3^ h^− 1^)	Y (g g^− 1^)	Reference
W29	batch	glucose	18	0.13	0.62	[18]
N15	batch	sunflower oil	150	1.32	1.56	[43]
NG40/UV7	batch	glycerol	115	0.6	0.64	[33]
187/1	batch	rapeseed oil	135	0.94	1.55	[53]
H222-S4(p67ICL1)T5	fed-batch	sucrose	140	0.73	0.82	[46]
A 101–1.31	fed-batch	glycerol	155	1.05	0.6	[35]
AWG7	repeated-batch	glycerol	154	0.94	0.6	[35]
AWG7	repeated-batch	glycerol	154	1.05	0.78	[46]
A101–1.22	repeated-batch	glycerol	124	0.85	0.6	[41]
SWJ-1b	batch	inulin	68.9	0.22	0,68	[30]
SWJ-1b 30	batch	inulin	84	0.39	0.84	[42]
AWG7	continuous	glycerol	97.8	0.49	0.98	[36]
AWG7 INU 5	repeated-batch	inulin	170	0.49^a^	0.61^b^	This study
AWG7 INU 8	repeated-batch	inulin	203	0.51^a^	0.85^b^	This study

^a^ the highest productivity noticed during the process^, b^ the highest yield noticed during the process

Q – productivity of citric acid, Y – yield of citric acid production

Additional advantage of the process with inulinase positive transformants of AWG7 strain growing on inulin is the low level of the undesired ICA (below 2 g dm^− 3^, Tables 2 and 3). The formation of ICA is strain and substrate dependent and differs among different cultivation systems [43]. In general, the concentration of ICA secreted by different wild strains of *Y. lipolytica* growing on carbohydrates or glycerol ranges from 8 to 16% [45, 46]. Other *Y. lipolytica* strain - Wratislavia K1, also derivative of Polish A-101 strain, during fed-batch culture on IN secreted 6.6–12.9 g dm^− 3^ [31]. The high CA/ICA ratio obtained in the RBC process with IN during the presented study will facilitate the process of its purification from the post-culture medium. The transformant AWG7 INU8 can produce 120 U g^− 1^ of inulinase activity. Liu et al. [30] determined the activity of immobilized inulinase with 6× His-tag to be 22.6 U mg^− 1^ of cell dry mass after 96 h of the cell growth for *Y. lipolytica* expressing the inulinase gene.

## Conclusion

In conclusion, the growing IN market and increasing amount of IN-rich wastes prompt to find new ways of their utilization. The presented study showed great potential of genetically engineered strains of *Y. lipolytica* AWG7 expressing inulinase to efficiently convert IN to highly desired CA. Furthermore, application of the RBC system allowed to increase the CA titer to 200 g dm^− 3^, which is, to our knowledge, the highest concentration of CA reported for *Y. lipolytica*.

## Methods

### Strains

The parental strain *Y. lipolytica* AWG7 used in this study was isolated from *Y. lipolytica* A-101-1.31 strain after its exposure to UV irradiation [47]. The strain belongs to the Yeast Culture Collection of the Department of Biotechnology and Food Microbiology, Wroclaw University of Environmental and Life Sciences, Poland. The strain has been deposited in a culture collection CIRM - Levures (INAG 36017) under number CLIB 81. This strain is a very efficient CA producer and is not able to form mycelium (data not shown). The new derivatives of AWG7 strain (AWG7 INU 1, AWG7 INU 2, AWG7 INU 3, AWG7 INU I, AWG7 INU 5, AWG7 INU 6, AWG7 INU 8) expressing the inulinase gene from *Kluyveromyces marxianus* CBS6432 (*INU1* gene; GenBank: X57202.1) were constructed according to the procedure described previously [31]. *Y. lipolytica* transformants were selected on the YPD medium with 400 μg cm^− 3^ of hygromycin B. Verification of the transformants was performed both, by PCR as well as growth detection on solid minimal medium with 1% of IN. Seven randomly selected transformants were chosen for further analysis of citric acid production in bioreactors. All yeast strains used in this study were stored at − 80 °C and refreshed before use by 24 h culture in YPD medium at 28 °C, 2.33 Hz.

### Media and culture conditions

IN (Mlyn Oliwski, Gdańsk, Poland), glucose and fructose (Sigma-Aldrich) were used as sources of carbon and energy. Inoculum medium consisted of glucose – 50 g, yeast extract − 3 g (Sigma-Aldrich), malt extract – 3 g (Sigma-Aldrich) and peptone – 5 g (Sigma-Aldrich) in distilled water – 1 dm^3^. The yeast were grown in 0.3-dm^3^ flasks containing 0.05 dm^3^ of the medium on a rotary shaker (CERTOMAT IS, Sartorius Stedim Biotech), at 30 °C, 140 rpm for 72 h, as described previously [48, 49]. In order to inoculate media for bioreactor experiments, 0.2 dm^3^ of inoculum (10% of the working volume) was used.

### Determination of IN utilization using microcultures

Inoculation medium (YPD) contained: glucose – 20 g, yeast extract – 10 g and bacteriological peptone – 20 g in 1 dm^3^ of distilled water. The inoculation cultures were grown for 24 h at 28 °C, 2.33 Hz on a rotary shaker (CERTOMAT IS, Sartorius Stedim Biotech).

The growth of *Y. lipolytica* AWG7 and its derivatives was analyzed in Bioscreen C system (Oy Growth Curves Ab Ltd., Finland), in Yeast Nitrogen Base medium (Sigma-Aldrich) supplemented with 2% (w v^− 1^) of glucose, fructose or IN. The overnight inoculation cultures were centrifuged and washed twice with sterile water. The yeast strains grew in 150 μl of the appropriate medium using 100-well microplates. The optical density (OD_600_) of the cells was standardized to 0.15. The experiments were performed in quintuples at 28 °C under constant, intensive agitation. The growth of cells was monitored by measuring the OD at 420–600 nm every 30 min for 48 h. Rakicka et al. [49] have published this methodology previously.

### CA biosynthesis in bioreactors

The differences in CA production among the 8 investigated strains were tested using batch cultures in medium containing: inulin – 100 g, NH_4_Cl – 2 g, KH_2_PO_4_–0.25 g, MgSO_4_ × 7H_2_O – 1.0 g, yeast extract – 1 g in 1 dm^3^ of tap water [35]. The parental strain (AWG7) was cultivated on glucose and fructose as a control experiment.

The intensification of CA production was performed by applying the repeated batch strategy (RBC) for only two best CA producers – AWG7 INU 5 and AWG7 INU 8 transformants. The medium used for CA biosynthesis was as described above. After the whole substrate was exhausted from the medium, 800 cm^3^ of the culture was withdrawn, and replaced by the same volume of fresh medium. This procedure was repeated three times (Fig. 3). During each RBC culture the working volume at the beginning of the process was maintained at 2 dm^3^. The end of each RBC cycle was determined when the concentration of fructose dropped below 20 g dm^− 3^.

All bioreactor cultures were performed in 5-dm^3^ stirred-tank reactor (BIOSTAT B-PLUS, Sartorius, Germany) with the working volume of 2.0 dm^3^ at 28 °C. Aeration and stirring rates were set at 0.6 m^− 1^ and 13.3 Hz, respectively. The pH 5.5 was maintained by additions of 5 M NaOH solution, as described previously [48, 50]. All cultures were cultivated until complete exhaustion of the carbon source. Bioreactor with the appropriate medium was autoclaved at 121 °C for 20 min. All cultures were conducted in two biological replicates.

### Analytical methods

Ten milliliters of culture broth was centrifuged (5 min, 2700 RCF). The biomass was washed twice with distilled water and filtered on 0.45 mm pore-size membranes. The biomass concentration was determined gravimetrically after drying at 105 °C and expressed in grams of cell dry weight per liter (g dm^− 3^). The concentration of substrate (glucose, fructose) and CA was measured in the supernatants by high-performance liquid chromatography (Dionex- Thermo Fisher Scientific, UK) using a Carbohydrate H+ Column (Thermo Scientific, Waltham, MA) coupled to a UV detector (k = 210 nm) and refractive index detector (Shodex, Ogimachi, Japan). The column was eluted with 25 mM trifluoroacetic acid at 65 °C and a flow rate of 0.6 cm^3^ min^− 1^. The concentration of isocitric acid (ICA) in the supernatant was determined using an Isocitrate Assay Kit (Sigma-Aldrich). This methodology has been described previously by Rakicka et al. [48, 50].

Inulinase activity of the strains was determined according to Gong et al. [51]. The reaction mixture containing 0.1 cm^3^ of the supernatant, 0.9 cm^3^ of phosphate buffer (0.1 M, pH 6.0) and 1% (mass per volume) of IN (Sigma) was incubated at 50 °C for 15 min. The reaction was inactivated immediately by keeping the reaction mixture at 100 °C for 10 min. The amount of reducing sugar in the reaction mixture was assayed by the method introduced by Miller [52]. One unit of inulinase activity (U) was defined as the amount of enzyme that releases 1 μM of reducing sugar per minute nder the assay conditions applied in this study. The specific inulinase activity was calculated as units per gram (U g^− 1^) of cell dry mass.

### Calculation of fermentation parameters and list of abbreviations

The yield of CA production from IN (Y), expressed in g g^− 1^, was calculated using the formula:

$$ Y=\frac{CA}{IN} $$

The productivity of CA in batch and repeated-batch culture (Q), expressed in g dm^− 3^ h^− 1^, was calculated as:

$$ Q=\frac{CA}{\varDelta t} $$

In all formulas, CA stands for citric acid concentration, IN stands for the consumed inulin concentration in the culture (g dm^− 3^), t is the duration of the fermentation process (h).

## Abbreviations

CA: Citric acid
ICA: Isocitric acid
IN: Inulin
RBC: Repeated batch culture
